# Is obesity a protective factor in pediatric trauma? A retrospective review in a tertiary trauma centre over the decade

**DOI:** 10.1007/s00383-025-06193-6

**Published:** 2025-09-17

**Authors:** Phoenix W. Y. Wong, Kenneth K. Y. Wong

**Affiliations:** Division of Paediatric Surgery, Department of Surgery, School of Clinical Medicine, LKS Faculty of Medicine, The University of Hong Kong, Hong Kong SAR, China

**Keywords:** Obesity, Trauma, Paediatric, Risk factors

## Abstract

**Purpose:**

The obesity paradox has been demonstrated in the adult trauma population. Yet there are conflicting results in children. The study aims to investigate the effect of childhood obesity on the outcomes after sustaining trauma.

**Method:**

All children (< 18-year-old) who attended a tertiary trauma centre from January 2010 to March 2022 due to impact trauma were included. The obese group was defined by a body weight > 95th percentile for age, and others were in the non-obese group. Outcomes, including injury-severity-scores (ISS), morbidity/mortality and length-of-stay (LOS), were compared between the groups. Subgroup analyses were performed on causes of injury and age groups. Severe injury was defined by ISS > 15.

**Results:**

148 subjects were included in the analysis (23 in the obese group; 123 in the non-obese group). They were similar in terms of injury causes and outcomes. The obese group had longer LOS (4 days vs 2 days, *p* = 0.022), and a higher proportion of them had severe injuries when < 11-year-old (28.6% vs 9%, *p* = 0.034).

**Conclusion:**

In contrast to adults, childhood obesity appears not to be a protective factor in trauma, and may be a risk factor, especially in younger children, for more severe injuries. Careful evaluation of obese children sustaining trauma is warranted to prevent injuries from being overlooked.

## Introduction

Trauma is the leading cause of pediatric mortality worldwide, with more than 10,000 deaths in the USA annually [1]. In Hong Kong, there are 3 pediatric deaths per 100,000 population [2]. The obesity paradox denotes a survival advantage for obese individuals despite an increase in morbidity after sustaining traumatic injuries [3]. This relationship has been well demonstrated in adults with thoracic and abdominal injuries, in which overweight and obese individuals enjoy a protective effect against mortality at the cost of longer hospitalization and ventilation days. Research in the pediatric population, on the other hand, is limited by inconsistent findings. While earlier literature has suggested an increased body mass index (BMI) percentile is associated with increased morbidity and mortality [4–6], the latest evidence suggests that the obesity paradox might exist in children [7, 8]. Possible reasons include cushion effect from adipose tissue and behavourial tendency to avoid risk-taking activities. Majority of the current literature was performed in the USA, where ballistic and penetrating injuries occur more frequently than in our locality.

Childhood obesity is also a growing pandemic in Hong Kong, with its incidence reaching up to 30% after the COVID-19 era [9]. The aim of the study is to evaluate the role of childhood obesity on their outcomes after sustaining trauma in our locality, and assess whether the obesity paradox can also be similarly demonstrated.

## Methods

### Study design

A retrospective analysis was performed for all patients younger than 18 years old who attended a tertiary trauma centre due to impact trauma from January 2010 to March 2022. Data were retrieved from the institutional database collected prospectively, including all patients who attended for trauma-related complaints via the Department of Accident and Emergency (AED). Individual admission body weights (BW) were retrieved via the electronic medical records. Those without a documented BW were excluded. Based on the BW percentile for age, patients were further stratified into the obese group (BW > 95th percentile) and the non-obese group (BW ≤ 95th percentile).

The primary outcomes were injury severity as defined by the injury severity scores (ISS). A severe injury is defined by ISS > 15. Secondary outcomes were selected, including the baseline demographics (age and gender), mechanisms and involved systems of injury, hospitalization course (need for operation, major operations, intensive care unit [ICU] admission, ICU stay duration and length of stay [LOS]) and complications.

Further subgroup analyses were performed for different age groups (age >  = 11 years old vs age < 11 years old) and types of injuries (road traffic accidents [RTA] vs fall injuries).

### Setting

Our centre provides tertiary referral trauma specialty services covering a network of hospitals, serving around 0.6 million people. It is one of the three designated paediatric trauma centres in our locality. Children sustaining trauma are either admitted to AED from the injury scene directly, or via secondary diversion from trauma units from other hospitals in the network [2, 10].

### Statistical analysis

Continuous and categorical variables were expressed as median with range and frequency with percentage, respectively. The Mann–Whitney U test was used for comparison of continuous variable, and Pearson’s chi-square test was used for comparing discrete variables. Statistical significance was denoted by *p*-value < 0.05. Statistical analyses and calculations were performed using the software Statistical Package for the Social Sciences (SPSS^®^; IBM, USA), Version 26.

## Results

717 children aged below 18 years old attended AED for trauma-related complaints. There were no isolated penetrating or ballistic injuries. Among them, 148 children (67.7% male, 32.4% female) who sustained impact trauma and had body weights documented were included in the analysis 72 attended in or before December 2016 while the rest attended after January 2017. 104 (70.3%) were younger than 11 years old. 41 children had RTA, while 107 suffered injuries from fall. 26 patients (17.6%) had severe injuries as defined by an ISS score greater than 15.

There were 123 children in the non-obese group and 25 in the obese group. As shown in Table 1, the two groups were similar in terms of background demographics, injury mechanisms, patterns, and severities (in terms of ISS, operation need, ICU admissions, and percentage of patients suffering from severe injuries). The proportion of obese to non-obese patients remained similar over the 12-year period in the study. The female-to-male ratios were 1:1.86 and 1:4 in the non-obese and obese groups, respectively. 62 patients (51 in the non-obese group and 11 in the obese group) had cross-sectional imaging as part of their management in the form of computer tomography (CT), 27 being performed between 2010 and 2016 and 35 done after January 2017. There were 3 mortalities and 2 trauma-related morbidities all within the non-obese group, but the differences were not statistically significant. Longer LOS was observed in the obese group (4 days vs 2 days, *P* = 0.022) (Table 2).

**Table 1:** Demographics, injury severity and pattern, and outcomes of obese and non-obese group

	Non-obese group (*n* = 123)	Obese group (*n* = 25)	*P* value
Age (years old)	5 (0–17)	9 (1–17)	0.032
< 11 years old	89 (72.4%)	14 (56%)	0.1
Male: female	80:43	20:5	0.133
Timing of injury			0.61
2010–2016	61 (84.7%)	11 (15.2%)	
2017–2022	62 (81.6%)	14 (18.4%)	
Injury mechanisms			0.285
RTA	32 (26%)	9 (36%)	
Fall	91 (74%)	16 (64%)	
Cross-sectional imaging	51 (41.5%)	11 (44%)	0.815
2010–2016	23 (37.7%)	4 (36.4%)	0.933
2017–2022	28 (45.2%)	7 (50%)	0.743
ISS	4 (1–59)	5 (1–36)	0.056
> 15	20 (16.3%)	6 (24%)	0.354
2010–2016	7 (11.5%)	2 (18.2%)	0.619
2017–2022	13 (21%)	4 (28.6%)	0.502
Operation performed	71 (57.3%)	16 (64%)	0.533
Major operations	44 (35.5%)	13 (52%)	0.121
ICU Admissions	31 (25%)	7 (28%)	0.754
Patterns of injuries			
Head	25 (20.2%)	6 (24%)	0.666
Thorax	13 (10.5%)	2 (8%)	0.707
Abdomen and pelvis	12 (9.7%)	3 (12%)	0.725
Extremities	44 (35.5%)	11 (44%)	0.421
Skin and soft tissue	59 (47.6%)	13 (52%)	0.687
Face	17 (13.7%)	3 (12%)	0.819
Mortality	3 (2.4%)	0	1
Complications	2 (1.6%)	0	1
LOS (days)	2 (1–72)	4 (1–49)	0.022

*RTA* road traffic accident, *ISS* injury severity score, *ICU* intensive care unit, *LOS* length of stay

**Table 2:** Demographics, injury severity and pattern, and outcomes of obese and non-obese group for patients < 11 years old (*n* = 103)

	Non-obese group (*n* = 89)	Obese group (*n* = 14)	*P* value
Age (years old)	4 (0–10)	5.5 (1–9)	0.09
Male: female	54:35:00	12:02	0.063
Injury mechanisms			0.969
RTA	13 (14.6%)	2 (14.3%)	
Fall	76 (85.4%)	12 (85.7%)	
ISS	4 (1–29)	4 (1–36)	0.14
> 15	8 (9%)	4 (28.6%)	0.034
Operation performed	52 (57.8%)	9(64.3%)	0.646
Major operations	28 (31.1%)	6 (42.9%)	0.383
ICU admissions	31 (25%)	5 (35.7%)	0.093
Patterns of injuries			
Head	15 (16.7%)	5 (35.7%)	0.093
Thorax	0	1 (7.1%)	0.135
Abdomen and pelvis	5 (5.6%)	2 (14.3%)	0.225
Extremities	27 (30%)	5 (35.7%)	0.667
Skin and soft tissue	43 (47.8%)	4 (28.6%)	0.179
Face	17 (18.9%)	3 (21.4%)	0.823
Mortality	0	0	
Complications	0	0	
LOS (days)	2 (1–12)	3 (1–49)	0.054

*RTA* road traffic accident, *ISS* injury severity score, *ICU* intensive care unit, *LOS* length of stay

Further subgroup analyses were performed with regard to mechanisms of injuries. In those with RTA, none of the obese patient had thoracic injuries (0 vs 34.4%, *P* = 0.04). Yet the obese and non-obese group showed no differences in terms of ICU admission, LOS, mortality or morbidity. For children with fall injuries, the obese group again showed a longer LOS (3.5 days vs 2 days, *P* = 0.015) without significant differences in injury severities (Table 3).

**Table 3:** Demographics, injury severity and pattern, and outcomes of obese and non-obese group for patients >  = 11 years old (*n* = 45)

	Non-obese group (*n* = 34)	Obese group (*n* = 11)	*P* value
Age (years old)	13 (11–17)	14 (11–17)	0.765
Male: female	26:08:00	08:03	0.802
Injury mechanisms			0.651
RTA	19 (55.9%)	7 (63.6%)	
Fall	15 (44.1%)	4 (36.4%)	
ISS	5 (1–59)	9 (1–26)	0.948
> 15	12 (35.3%)	2 (18.2%)	0.287
Operation performed	19 (55.9%)	7 (63.6%)	0.651
Major operations	16 (47.1%)	7 (63.6%)	0.339
ICU Admissions	14 (41.2%)	2 (18.2%)	0.166
Patterns of injuries			
Head	10 (29.4%)	1 (9.1%)	0.173
Thorax	13 (38.2%)	1 (9.1%)	0.07
Abdomen and pelvis	7 (20.6%)	1 (9.1%)	0.386
Extremities	17 (50%)	6 (54.5%)	0.793
Skin and soft tissue	16 (47.1%)	9 (81.8%)	0.044
Face	0	0	
Mortality	3 (8.8%)	0	0.565
Complications	2 (5.9%)	0	1
LOS (days)	4 (1–72)	5 (2–13)	0.99

*RTA* road traffic accident, *ISS* injury severity score, *ICU* intensive care unit, *LOS* length of stay

Subgroup analyses were also performed regarding different age groups. In those who were younger than 11 years old, we observed that there was a greater proportion in the obese group who suffered from severe injuries when compared with the non-obese group (28.6% vs 9%, *P* = 0.034). This difference was not seen in those >  = 11 years old. LOS were comparable between the obese and the non-obese group regardless of age groups (Table 4).

**Table 4:** Demographics, injury severity and pattern, and outcomes of obese and non-obese group for patients with road traffic accident (*n* = 41)

	Non-obese group (*n* = 32)	Obese group (*n* = 9)	*P* value
Age (years old)	12 (0–17)	12 (6–17)	0.793
< 11 years old	13 (40.6%)	2 (22.2%)	0.311
Male: female	23:09	06:03	0.762
ISS	4.5 (1–50)	9 (1–26)	0.889
> 15	11 (34.4%)	2 (22.2%)	0.489
Operation performed	15 (46.9%)	7 (77.8%)	0.14
Major operations	12 (37.5%)	6 (66.7%)	0.119
ICU Admissions	16 (50%)	2 (22.2%)	0.138
Patterns of injuries			
Head	11 (34.4%)	1 (11.1%)	0.175
Thorax	11 (34.4%)	0	0.04
Abdomen and pelvis	4 (12.5%)	2 (22.2%)	0.597
Extremities	12 (37.5%)	5 (55.6%)	0.331
Skin and soft tissue	21 (65.6%)	6 (66.7%)	0.954
Face	2 (6.3%)	0	1
Mortality	2 (6.3%)	0	1
Complications	2 (6.3%)	0	1
LOS (days)	5 (1–48)	4 (2–13)	0.631

ISS injury severity score, *ICU* intensive care unit, *LOS* length of stay

## Discussion

Childhood obesity is a growing pandemic on both local and global scales. The incidence has further surged after the COVID era [9]. With an increasing population of overweight and obese children, it is important that clinicians recognize its impact on the outcomes in trauma. The “obesity paradox” suggests that obese individuals enjoy a survival benefit at the cost of increased morbidity [3, 8]. Of note, a large proportion of the literature published on the topic is done in the United States of America. The mechanisms of pediatric trauma injuries in our locality are quite different. The incidences of penetrating and gunshot injuries are drastically lower due to a lack of private gun ownership policy and differences in social culture. Our study here attempts to demonstrate whether the obesity paradox can be replicated in our East Asian locality (Table 5).

**Table 5:** Demographics, injury severity and pattern, and outcomes of obese and non-obese group for patients with fall injury (*n* = 107)

	Non-obese group (*n* = 91)	Obese group (*n* = 16)	*P* value
Age (years old)	4 (0–17)	6.5 (1–15)	0.098
< 11 years old	76 (83.5%)	12 (75%)	0.411
Male: female	56:35:00	14:02	0.044*
ISS	4 (1–59)	4.5 (1–36)	0.085
> 15	9 (9.9%)	4 (25%)	0.088
Operation performed	56 (61.5%)	9 (56.3%)	0.69
Major operations	32 (35.2%)	7 (43.8%)	0.511
ICU Admissions	14 (15.4%)	5 (31.3%)	0.126
Patterns of injuries			
Head	14 (15.4%)	5 (31.3%)	0.126
Thorax	2 (2.2%)	2 (12.5%)	0.105
Abdomen and pelvis	8 (8.8%)	1 (6.3%)	0.736
Extremities	32 (35.2%)	6 (37.5%)	0.857
Skin and soft tissue	37 (40.7%)	7 (43.8%)	0.817
Face	15 (16.5%)	3 (18.8%)	0.823
Mortality	1 (1.1%)	0	1
Complications	0	0	1
LOS (days)	2 (1–72)	3.5 (1–49)	0.015*

*ISS* injury severity score, *ICU* intensive care unit, *LOS* length of stay

* denotes p<0.05 and statistically significant

BMI is the most universally accepted modality for the assessment of obesity and overweight. Its use in children is being increasingly challenged for the overdiagnosis of childhood obesity. Emerging studies have shown that an elevated BMI in childhood reflects growing lean muscle mass instead of fat mass. In the setting of impact injuries, the body weights/masses play a vital role in impact injuries as they affect the reaction and collision forces. We therefore have opted to use the body weight percentile-for-age to define the obese and non-obese group due to the reasons above.

From our results, obesity appeared not to be protective against sustaining severe trauma nor death in children. On reviewing our database, there was no isolated penetrating injury in the series. In addition, the median BMI of obese Hong Kong children is much, much lower than that counterparts in USA [11–13]. Hence, the role of subcutaneous fat as an “urban armour” in reducing the severity of penetrating injuries appears to be less vital. The longer LOS seen in the obese group despite no differences in terms of ISS and proportion of severe injuries is similar to previous findings [6]. A possible explanation is that obesity leads to hindered mobility and slower rehabilitation after the index injury. Furthermore, no significant differences in overall complications were demonstrated between obese and non-obese groups in our cohort, in contrary to earlier publications. This may be attributed to the small sample size in this study. A multi-centre study with a larger sample size should be pursued to evaluate the effect of obesity on outcomes in pediatric trauma.

Subgroup analyses were performed for different age groups and mechanisms of injury. These two variables were selected as they were closely associated with the magnitude of trauma sustained in children. Age plays a vital role in pediatric trauma management, as ISS increases with age, the number of trauma increases in adolescents, and the mechanisms of injuries also vary among different age groups. Adolescents were at risk, in particular, most likely due to increased independence from caregivers, increased mobility, as well as risk-taking behaviour in puberty [2, 14, 15]. Our initial hypothesis was that older obese children may be at risk of sustaining severe injuries due to the reasons above. The age of 11, which has been used on many occasions, such as gonadal malignancies, as a surrogate for puberty onset, was chosen as the cut-off in our analysis. The obese and non-obese groups showed no differences in terms of the proportion of severe injuries for adolescents, while a higher proportion of younger obese children had an ISS > 15. We postulate that this was due to decreased mobility and sedentary lifestyle associated with obese children. In obese adolescence with increasing self-awareness of body image, they may have active avoidance in social activities and high-risk physical activity, with less time spent on the streets. In younger obese children, their mobility is further compromised due to a smaller physique, yet they may still enjoy time spent in parks and on the streets. Severe injuries could happen in this cohort when the caregiver was not alert or was distracted.

Another important constituent in the obesity paradox was a decreased incidence of thoracic injuries in the obese individuals in trauma [3, 8]. In analyzing the patterns of injuries, fewer thoracic injuries were shown in the RTA subgroup but not in the overall cohort or other subgroups. Blunt thoracic trauma in children carries a distinct pattern from adults, in which they occur less frequently in an isolated manner due to anatomical difference, but is more likely to be associated with morbidity and mortality [16]. However, fewer thoracic injuries did not translate into a change in management or overall outcomes, namely the need for operation, ICU admissions, complications and mortality in our cohort. Several reasons may be accountable for the result. First, our locality remains a relatively child-safe environment with only half the rate of unintentional child fatality when compared with the USA [2, 17]. Isolated thoracic injuries are rare in the absence of penetrating and ballistic injuries. Second, the majority of impact trauma in this cohort were low impact ones which resulted in relatively minor thoracic injuries, including small pneumothorax, hemothorax and lung contusion, etc. Almost all thoracic injuries included in the analysis were managed conservatively and/or with bedside procedures, such as bronchoscopy and chest drain insertion. Thus, the presence of thoracic injuries appears not to be a major determinant of outcomes in our locality.

A major pitfall in the study is the increasing frequency of using cross-sectional imaging in pediatric patients sustaining trauma-related injuries over the 12-year study period, and it may lead to a concern of a falsely elevated ISS from the detection of clinically occult injury on imaging. Though cross-sectional imaging in the form of computer tomography (CT) was not part of our centre’s trauma management protocol, nonetheless it was performed more liberally, as shown in Table 1 in which a greater percentage of children received CT when admitted in the second half of the study period for both the obese and the non-obese groups. Interestingly, the obese group had a larger proportion of patients who had severe injuries across the entire study period, despite a similar rate at imaging with the non-obese group. Yet there were no significant differences in terms of the clinical outcomes as mentioned above. Two issues were identified from this observation. First, a more liberal use of imaging alone may not be accountable for the increased proportion of severe injuries observed in the obese children in the cohort, and other factors in the obese group, such as decreased mobility and behavourial difference should be taken into consideration. Secondly, it was debatable whether a higher ISS would lead to a change in actual outcomes. Other parameters, such as the need for avoidance of strentuous exercise, repeated imaging and follow-up, were not measured in the current database. Further study will be needed to evaluate the quality of life of the patients post-injury.

Being a retrospective single-centre study, the generalizability of our result would be limited. Those who did not have a documented body weight and/or did not seek medical help in our centre due to minor injuries were not included in the analysis, which may introduce selection bias. Details on trauma mechanisms such as vehicle speed and patients’ role as pedestrians or passengers in RTA, and height of fall injury were not included in the analysis largely due to the small sample size as well as unclear documentation in the electronic medical records and trauma database. A proper and detailed documentations of medical records should be pursued in the future. We also observed that boys compromised a significant proportion in our study, which aligned with previous published findings due to possible behavioural differences between the gender [2]. Of note, the obese group showed an even lower female-to-male ratio, which may be explained by a much higher prevalence of obesity in boys from our local studies [11, 13].

Despite these limitations, our study has shone light on the questionable protective role of obesity in pediatric trauma. It is understandable that the role of physical examination may be limited due to the presence of abundant subcutaneous fat in obese children. Clinicians should have a higher level of suspicion when it comes to the care of these children to avoid overlooking underlying traumatic injuries. Imaging should be considered if physical examination findings were in discordance with patient’s complaint.

## Conclusion

Childhood obesity appears not to be a protective factor in children sustaining trauma, and may be a risk factor in young children for more severe injuries. Careful evaluation of obese children sustaining trauma is warranted to prevent injuries from being overlooked.

## Data Availability

No datasets were generated or analysed during the current study.

## References

[1] McFadyen JG, Ramaiah R, Bhananker SM (2012) Initial assessment and management of pediatric trauma patients. Int J Crit Illn Inj Sci 2(3):121–127 23181205 10.4103/2229-5151.100888 PMC3500003

[2] Chow CB et al (2019) Epidemiology of paediatric trauma in Hong Kong: a multicentre cohort study. Clin Epidemiol Glob Health 7(1):71–78

[3] Dvorak JE et al (2020) The obesity paradox in the trauma patient: normal may not be better. World J Surg 44(6):1817–1823 32006135 10.1007/s00268-020-05398-1 PMC7222933

[4] Rana AR et al (2009) Childhood obesity: a risk factor for injuries observed at a level-1 trauma center. J Pediatr Surg 44(8):1601–1605 19635312 10.1016/j.jpedsurg.2008.11.060 PMC3717372

[5] Ross PA et al (2016) Obesity and mortality risk in critically ill children. Pediatrics 137(3):e20152035 26908670 10.1542/peds.2015-2035

[6] Witt CE et al (2017) Obesity in pediatric trauma. J Pediatr Surg 52(4):628–632 27914588 10.1016/j.jpedsurg.2016.11.037 PMC5386804

[7] Ward SL et al (2016) Impact of weight extremes on clinical outcomes in pediatric acute respiratory distress syndrome. Crit Care Med 44(11):2052–2059 27355525 10.1097/CCM.0000000000001857 PMC5199718

[8] Carlson JP, Pena K, Burjonrappa S (2024) The obesity paradox in the pediatric trauma patient. J Pediatr Surg 59(2):275–280 37993398 10.1016/j.jpedsurg.2023.10.038

[9] Yip KM et al (2024) Physical fitness and body mass index status of Hong Kong primary schoolchildren across the COVID-19 pandemic, before and after school closure. J Pediatr 264:113729 37722554 10.1016/j.jpeds.2023.113729

[10] Tsang JT et al (2024) Epidemiological changes in the pattern of children’s traumatic injuries at Hong Kong emergency departments during the COVID-19 pandemic: a retrospective, single-institutional, serial and comparative study. Pediatr Surg Int 40(1):192 39012503 10.1007/s00383-024-05772-3 PMC11252212

[11] Ko GT et al (2008) The problem of obesity among adolescents in Hong Kong: a comparison using various diagnostic criteria. BMC Pediatr 8:10 18315886 10.1186/1471-2431-8-10 PMC2276189

[12] Tsoi MF et al (2022) Prevalence of childhood obesity in the United States in 1999–2018: a 20-year analysis. Obes Facts 15(4):560–569 35358970 10.1159/000524261 PMC9421675

[13] Wang JJ, Gao Y, Lau PWC (2017) Prevalence of overweight in Hong Kong Chinese children: its associations with family, early-life development and behaviors-related factors. J Exerc Sci Fit 15(2):89–95 29541138 10.1016/j.jesf.2017.10.001 PMC5812875

[14] Kuok CI, Chan WKY, Kwok AWL (2021) What and who should we focus in pediatric injury prevention - an analysis of critical pediatric trauma in a major trauma center in Hong Kong. Pediatr Neonatol 62(6):620–627 34330685 10.1016/j.pedneo.2021.05.027

[15] Tracy ET et al (2013) Pediatric injury patterns by year of age. J Pediatr Surg 48(6):1384–1388 23845634 10.1016/j.jpedsurg.2013.03.041 PMC4336172

[16] Alemayehu H, Aguayo P (2015) Pediatric blunt thoracic trauma. J Pediatr Intensive Care 4(1):35–39 31110848 10.1055/s-0035-1554987 PMC6513125

[17] West BA et al (2021) Unintentional injury deaths in children and youth, 2010–2019. J Safety Res 78:322–330 34399929 10.1016/j.jsr.2021.07.001 PMC11428120

